# Preattentive Processing of Numerical Visual Information

**DOI:** 10.3389/fnhum.2017.00070

**Published:** 2017-02-17

**Authors:** Philipp N. Hesse, Constanze Schmitt, Steffen Klingenhoefer, Frank Bremmer

**Affiliations:** ^1^Department of Neurophysics, Philipps-Universität MarburgMarburg, Germany; ^2^Center for Molecular and Behavioral Neuroscience (CMBN), Rutgers UniversityNewark, NJ, USA

**Keywords:** visual mismatch negativity, vMMN, number, subitizing-range, preattentive

## Abstract

Humans can perceive and estimate approximate numerical information, even when accurate counting is impossible e.g., due to short presentation time. If the number of objects to be estimated is small, typically around 1–4 items, observers are able to give very fast and precise judgments with high confidence—an effect that is called subitizing. Due to its speed and effortless nature subitizing has usually been assumed to be preattentive, putting it into the same category as other low level visual features like color or orientation. More recently, however, a number of studies have suggested that subitizing might be dependent on attentional resources. In our current study we investigated the potentially preattentive nature of visual numerical perception in the subitizing range by means of EEG. We presented peripheral, task irrelevant sequences of stimuli consisting of a certain number of circular patches while participants were engaged in a demanding, non-numerical detection task at the fixation point drawing attention away from the number stimuli. Within a sequence of stimuli of a given number of patches (called “standards”) we interspersed some stimuli of different numerosity (“oddballs”). We compared the evoked responses to visually identical stimuli that had been presented in two different conditions, serving as standard in one condition and as oddball in the other. We found significant visual mismatch negativity (vMMN) responses over parieto-occipital electrodes. In addition to the event-related potential (ERP) analysis, we performed a time-frequency analysis (TFA) to investigate whether the vMMN was accompanied by additional oscillatory processes. We found a concurrent increase in evoked theta power of similar strength over both hemispheres. Our results provide clear evidence for a preattentive processing of numerical visual information in the subitizing range.

## Introduction

Our daily lives are full of situations that require the processing of numerical information. We have to decide, for example, on the number of stripes that brand our new pair of running shoes and, after the purchase, rate their quality using a scale of 1–5 stars. Similarly, non-human primates and other animals might have to judge which of the two branches of a tree carries more fruit and thus should be foraged first. Given the amount and importance of numerical information that is relevant for the survival in modern and more ancient worlds, it should come as no surprise that the ability to make use of non-symbolic numerical information has not only been observed in human adults, but also in prelinguistic children, non-human primates and other animals including birds, dogs and fish (Dehaene, 2011).

Although still debated (see e.g., Durgin, 2008; Dakin et al., 2011; Gebuis and Reynvoet, 2012), the notion of a genuine “sense of number”, i.e., an intuitive understanding of set sizes and relations, has gained some evidence in recent years (Nieder, 2005; Burr and Ross, 2008; Ross and Burr, 2010; Dehaene, 2011; Cicchini et al., 2016). In this view, estimating which branch carries more fruit is not an intricate cognitive thought process, but is actually surprisingly similar to the processing of other basic visual properties like orientation or color.

In line with this, it has been shown behaviorally that numerosity, similar to other sensory features, is subject to adaptation (Burr and Ross, 2008) and obeys Weber’s law in large parts (Revkin et al., 2008).

In the same vein, genuine representations of numerosity have been identified on the single neuron level in the posterior parietal and prefrontal cortex of the macaque monkey (Nieder et al., 2002, 2006; Sawamura et al., 2002; Roitman et al., 2007). Importantly, these neurons possess tuning properties similar to other basic sensory features, are robust to variations in low level visual features, and exist in animals that had never been trained in numerical tasks (Viswanathan and Nieder, 2013).

In the human brain, using fMRI, the parietal cortex has also been shown to be responsive to numerosity (Dehaene et al., 1999; Pinel et al., 1999; Piazza et al., 2004) and, interestingly, to yield a robust topographic representation of it (Harvey et al., 2013).

While the aforementioned studies can be considered as evidence that numerosity might indeed be a rather low level visual feature in the primate visual system, recent studies on preattentive processing, which is another characteristic property of low level sensory processes, have provided mixed results. It is well established that humans are able to instantaneously see and report the exact number of elements in visual sets of small numerosity (like the set of stripes on your running shoes for example). This effect is known to exist for sets that contain up to three or four items and has been named subitizing (Jevons, 1871; Kaufman et al., 1949; Mandler and Shebo, 1982). Due to the speed and the subjective ease of the phenomenon, subitizing has traditionally been assumed to be preattentive (Trick and Pylyshyn, 1993, 1994; Pylyshyn, 2001), i.e., to happen even when subjects are not involved in a numerosity task and when their focus of spatial attention is not directed towards the stimulus. More recent studies (Egeth et al., 2008; Olivers and Watson, 2008; Railo et al., 2008; Anobile et al., 2012) have challenged this view and reported that subitizing is influenced by attentional load.

An established electrophysiological measure to unveil preattentive processing is the *mismatch negativity (MMN)* response in EEG. MMN describes the phenomenon that *event-related potentials* (ERPs) differ, when within a sequence of equal stimuli (called standards) a deviant stimulus (oddball) occurs. Typically, ERP-amplitudes between 100 ms and 250 ms evoked by the standard stimuli are less negative than ERP-amplitudes elicited by the deviant stimuli. The MMN became an established marker to investigate preattentive processing, because it can be observed even when participants are engaged in a difficult task drawing attention away from the relevant stimulus feature or its spatial location (for a review, see e.g., Sussman, 2007).

MMN responses have initially been reported for various features of auditory stimuli, among them: frequency, loudness (e.g., Näätänen et al., 1978), duration (e.g., Jacobsen and Schröger, 2003) and recently also numerosity (Ruusuvirta and Astikainen, 2016).

By now, MMN responses have also been demonstrated in the visual domain, termed visual MMN (vMMN; for a review, see Kimura, 2012), including features such as color (e.g., Czigler and Sulykos, 2010; Müller et al., 2010), size (e.g., Kimura et al., 2008a), orientation (e.g., Astikainen et al., 2008; Czigler and Sulykos, 2010; Kimura et al., 2010), location (e.g., Berti, 2009), shape (e.g., Bubrovszky and Thomas, 2011), luminance/contrast (e.g., Kimura et al., 2008b), spatial frequency (e.g., Kenemans et al., 2003), direction of motion (e.g., Pazo-Alvarez et al., 2004; Amenedo et al., 2007), duration (e.g., Qiu et al., 2011) and facial expression (e.g., Zhao and Li, 2006).

Important for our current study, a number of EEG studies have reported modulations of various ERP components by numerosity. Usually the effects were observed in the time range between 150 ms and 250 ms after stimulus onset (Libertus et al., 2007; Hyde and Spelke, 2009, 2012; Park et al., 2016), but modulations beginning as early as 75 ms have also been reported (Park et al., 2016). While some of the earlier results have been attributed to confounding contributions of low level visual properties (Gebuis and Reynvoet, 2012, 2013), recent findings demonstrate that the neuronal mechanisms underlying numerosity processing do indeed generate strong signals that can be measured in the EEG (Park et al., 2016). Using a regression-based approach, Park et al. (2016) could show that ERPs between 150 ms and 250 ms over parieto-occipital electrodes are actually more sensitive to modulations in numerosity than to other low level visual properties. So far, to the best of our knowledge, no EEG study has investigated the MMN as a marker of preattentive processing of numerosity in the visual domain.

In our study, we hypothesized MMN responses in the subitizing range in response to non-symbolic visual stimuli. In order to test for this hypothesis, we presented sequences of visual stimuli, consisting of a certain number of circular patches (1, 2 or 3), to our subjects. As in classical MMN paradigms, standard stimuli consisting of a certain number of patches were presented frequently in a particular sequence while a few deviant stimuli, consisting of a different number of patches, were interspersed from time to time. Subjects were engaged in a difficult foveal detection task guiding their attention off the actual stimuli that were presented in the periphery of the visual field. To avoid potential confounds because of physical differences in the stimuli, we compared conditions of visually identical stimuli that had been presented as standard stimulus in one condition and as oddball in another.

Our results clearly revealed the presence of vMMN responses for infrequent numerosity changes. Hence, our results provide further evidence for the idea that the processing of numerical information in the subitizing range contains a preattentive component.

## Materials and Methods

### Participants

A total of 10 participants (five male) aged between 22 and 30 (mean 26.9) were recruited from the university population. All participants had normal or corrected to normal vision. All participants except two (subject I, author PNH and subject II, author CS) were naïve about the purpose of the study and were compensated with 8 € per hour for their participation. After completing the full experiment each interested participant was given full disclosure on the purpose of the experiment. This study was carried out in accordance with the recommendations of and approved by the Ethics Committee of the Faculty of Psychology at Philipps-University Marburg, and conducted according to the principles of the Declaration of Helsinki. Participants provided written informed consent before commencing the experiment.

### Setup

Experiments were performed in a dark, sound attenuated and electrically shielded room. Participants sat on a chair resting their heads on a chin rest, placed centrally in front of a screen. The distance between the screen and the participants’ eyes was 68 cm. The screen was a 52 cm (41.8°) wide and 29.25 cm (24.3°) high TFT monitor (ViewPixx/3D Lite, VPixx Technologies Inc., Saint-Bruno, QC, Canada). The resolution of the screen was set to 1920 × 1080 pixels and the refresh rate to 100 Hz. By employing the monitor’s *scanning-backlight-mode* the behavior of a CRT screen was simulated. Stimulus presentation was controlled by the Psychophysics Toolbox 3 (Brainard, 1997; Pelli, 1997; Kleiner et al., 2007). EEG was recorded continuously (sample rate: 1000 Hz) using 64 Ag/AgCl active electrodes (Brain Products GmbH, Gilching, Germany). Electrode scalp locations conformed to the extended international 10–20 system. Electrode impedance was kept below 5 kΩ. EEG signals were recorded with Brain Vision PyCorder. Participants’ right eye position was recorded with an EyeLink 1000 Plus (SR Research Ltd., Ottawa, ON, Canada) at a sampling rate of 1000 Hz and further processed as additional EEG-channels (horizontal and vertical gaze position on the screen).

### Stimuli

**Figure 1:**
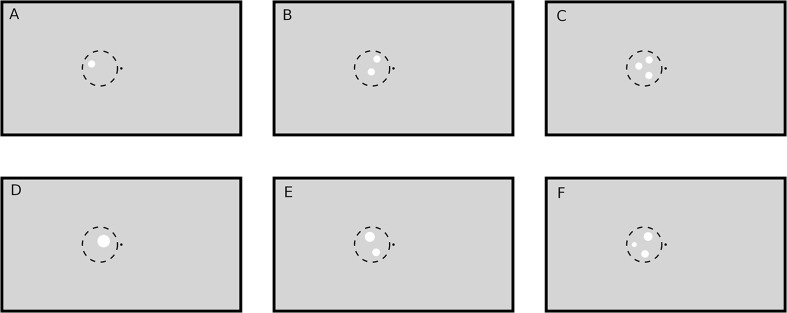
**Example stimuli (not drawn to scale).** A black point (luminance: 0.2 cd/m^2^) in the middle of the screen on a gray (luminance: 8.6 cd/m^2^) background served as fixation target. Stimuli were white circular patches (luminance: 62 cd/m^2^) drawn in the left or right (not shown here) visual hemifield on the vertical meridian (eye level). The one **(A,D)**, two **(B,E)** and three **(C,F)** circular patches were pseudorandomly placed within an imaginary circle with radius 3.3° and bound to some other constraints (see text). Circular patch size was either constant (condition SizeCon: **A–C**) or varied by keeping the total stimulus area (and hence luminance) constant (condition LumCon: **D–F**).

A black fixation point (luminance: 0.2 cd/m^2^; radius: 0.11°) in the middle of a gray screen (luminance: 8.6 cd/m^2^) was displayed throughout all trials. As target for the subjects’ task a small ring (0.03° thick, inner radius 0.11°) colored in dark gray (luminance: 1 cd/m^2^) was occasionally displayed around the fixation point, causing the impression of an enlargement of the central fixation point. The parameters of the target ring were optimized in pilot runs before the actual experiments such that the task could only be accomplished with high accuracy when the subjects paid close attention to the fixation target. The task-unrelated numerosity stimuli consisted of one, two or three white circular patches (luminance: 62 cd/m^2^). Patches could be presented either in the left or the right visual hemifield and had either the same radius or the same total area, so that either patch size (hereafter called “SizeCon”) or total luminance (hereafter called “LumCon”) was conserved (Figure 1). In the following we will use the terms “Right” and “Left” to refer to the condition in which the stimuli were presented in the right part of the visual field, or in the left part, respectively. *X* and *Y* coordinates of the center of the patches within a visual hemifield were pseudorandomly chosen beforehand (from a uniform distribution). They were located within an imaginary circle with radius 3.3° and center position 3.9° to the left or to the right of the fixation point, which was presented on the vertical meridian at eye level. In condition SizeCon, all circular patches had a radius of 0.65°. In the condition LumCon, the absolute area of the presented circular-patches was set to 8000 pixels ±2% (uniformly distributed jitter). The radiuses were chosen pseudorandomly in a manner, that no radius would differ more than ±40% from the mean radius and that no radius would be smaller than 0.22°. Distance between patches was at least 0.39°.

### Procedure

**Figure 2:**
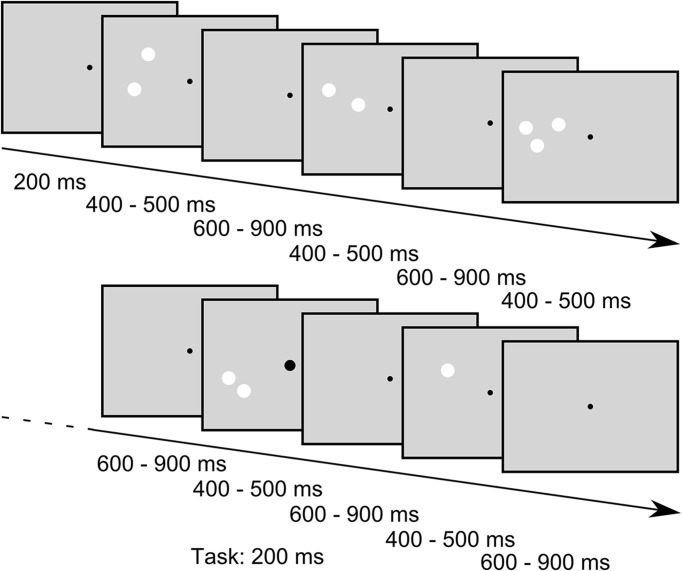
**Example stimulus sequence of the SizeCon condition (not drawn to scale).** Subjects were fixating a central dot and were asked to report small enlargements of the fixation point that occurred occasionally while ignoring the task irrelevant peripheral stimuli. The fixation point was presented throughout the trials while the peripheral stimuli were flashed for random durations between 400 ms to 500 ms with inter stimulus intervals lasting between 600 ms and 900 ms. Sequences of “standard” stimuli, i.e., stimuli that all had the same number of patches, were interspersed with stimuli of different numerosity (“oddballs”, presented in 20% of the trials); for further details of the stimuli see main body text.

While the subjects were engaged in a task that required the detection of enlargements of the central fixation point (see below), we presented sequences of stimuli consisting of different numbers of white circular patches in the periphery. In one sequence we always presented 30 stimuli in a row. In the following we will refer to these sequences as “blocks” that are separated into “trials” with each trial comprising the presentation of one stimulus. In a trial the stimulus was presented 200 ms after trial onset and was presented for a random duration between 400 ms to 500 ms. The trials ended after a random time between 400 ms to 700 ms after stimulus offset (for a schema of stimulus presentation, see Figure 2). Jitters in stimulus duration and inter stimulus interval were pseudorandomly chosen from a uniform distribution. In each block one standard-amount of patches (one, two or three) was presented in 24 trials (80%) while the remaining two quantities were presented in three trials (10%) each, resulting in an oddball-ratio of 1:4. If, for example, the quantity one was the standard stimulus in a block, the quantities two and three were considered deviants and were thus presented in three trials of the block each. Importantly, in this presentation scheme each standard stimulus also served as deviant stimulus in the two other conditions, thus allowing the comparison of the EEG response to physically identical stimuli that only differed by their role as standard or deviant within a block. Further constraints in the presentation order of the stimuli were that the first four trials in a block were always standard trials, and a deviant trial was always followed by a standard trial. Within a given block of trials, the stimulus position (left or right part of the visual field) and the conserved feature (SizeCon or LumCon) always remained the same. One experimental set consisted of 24 blocks, each condition (*standard value* (3) × *position* (2) × *conserved feature* (2)) was presented twice. Hence, in one set 720 trials were performed. The order of blocks was pseudorandomly distributed within a set. Between blocks an Eyelink drift correction was performed to recalibrate the recorded eye position signal, but also to provide a breather to our subjects. Participants decided themselves when to start the next block of trials. Every participant performed a total of 18 sets on three different days (not necessarily subsequent). Data from one subject were collected on 4 days due to interim hardware problems of the experimental setup.

In trials with a task-condition (72 trials = 10%) the fixation point became larger for 200 ms at a pseudorandomly chosen point in time between 100 ms after beginning of the trial and 150 ms before the end of the trial (uniformly distributed). Responses made between 300 ms and 800 ms after the actual target enlargement were considered as correct. The experiment was designed in a manner that task-conditions appeared solely in standard trials. Participants were instructed to fixate the fixation point and press a key (key down on a standard-keyboard) as fast as possible, whenever the diameter of the fixation point was increased. Furthermore, subjects were told to ignore the flashed stimuli and try to reduce blinks during a block of trials.

### Analysis

The participants’ behavior in the task, which was indicated by button presses as response to an increase of the diameter of the fixation point, was evaluated by determining the percentage of correctly pressed keys compared to all task-condition trials. Furthermore, the false alarms, as well as button presses outside 300–800 ms after task onset, were detected.

#### Preprocessing

The EEG data were evaluated offline using Brain Vision Analyzer, MATLAB and R. In a first step of our data analysis, the mean value of the mastoids (TP9 and TP10) was chosen as a new reference. Then a bandpass filter (second-order, zero phase shift Butterworth filter with cutoff frequencies 0.5 Hz and 40 Hz) was applied, to reduce noise (such as influences of 50 Hz mains frequency). These filtered, continuous data were temporally sliced into individual trials, starting 200 ms before stimulus onset and ending 500 ms after stimulus onset. Trials with button presses (correct and incorrect) were removed from further analysis, as were trials in which a button press would have been required, but was not performed. The mean signal amplitude of each trial in the time window from −110 ms up to 0 ms before stimulus onset was subtracted from the entire signal of the trial, separately for each electrode (baseline correction). In a global automatic artifact rejection all trials in which the signal of any electrode exceeded a difference of ±100 μV within an interval of 100 ms were excluded from further analysis. In a last step, trials with eye movement artifacts, such as blinking or breaking fixation, were automatically removed. Precise timing of stimulus presentation was controlled by a photosensitive-diode, being invisible to the subjects, attached to the screen.

#### Statistical Analyses

Analyses were performed with two different methods. We performed: (i) a “classical” MMN analysis based on ERPs; and (ii) a *time-frequency-analysis* (TFA) on the other hand. Trial selection and data preprocessing did not differ between both analyses.

For each subject, trial data were sorted by conditions (*presentation side × conserved feature × number × deviance*) and averaged per condition separately for ERP-analysis and TFA. Importantly, in the TFA the averaging was done only after the single trial data had been transformed into the time-frequency domain first (see below). Our pre-analysis, as described above, might have led to variations in the number of available trials between participants or conditions. The averaging, however, aimed to minimize variations in performance (blinks, breaks of fixation, button presses and bad electrode signals) and guaranteed that data from each participant and each condition contributed equally to the results.

#### Event Related Potentials Analysis

Based on results of vMMN studies (for a review, see e.g., Kimura, 2012) and the reported effects of number on visual ERPs (Plodowski et al., 2003; Libertus et al., 2007; Hyde and Spelke, 2009; Hyde and Wood, 2011), we expected a vMMN concerning numerosity to occur on parieto-occipital electrodes contra-lateral to the presentation side of the stimulus. Hence, we confined our analysis to EEG-data from electrodes P6, P8 and PO8 (i.e., from over the right cortical hemisphere) for all *Left* conditions and data from P5, P7 and PO7 for all *Right* conditions. The temporal characteristics of vMMN effects have been shown to reveal some variation across studies and seem to be dependent on the exact experimental condition (see Pazo-Alvarez et al., 2003 for a review). Hence, in a first step of our statistical analysis, we aimed to identify the time of occurrence of the MMN by using a statistical method proposed by Guthrie and Buchwald (1991).

As a first step, we down-sampled our data from 1000 Hz to 200 Hz by averaging over five consecutive samples. Further analysis was performed on this down-sampled data set within a time interval from 120 ms to 240 ms after stimulus onset. Hence, each test interval consisted of 24 samples. Single-sided *t*-tests across the participants were performed on ERP-differences (deviant condition−standard condition) averaged within the participants. A test whether statistically this difference was significantly different from and below zero was performed separately for each presentation side (Left vs. Right) and conserved feature (SizeCon vs. LumCon). This procedure resulted in 24 *p*-values for each condition, one for each tested sample. In order to correct for multiple comparisons and to account for potential covariance in time in the data, the method of Guthrie and Buchwald (1991) relies on the first order autocorrelation coefficient (Φ) of the data set under investigation. Depending on the value of the autocorrelation coefficient, a threshold on the number of consecutive samples with a *p* < 0.05 can be defined; if the number of consecutive samples that are individually significant exceeds this threshold, the corresponding segment of the time series can be considered significant as a whole (see Guthrie and Buchwald, 1991, Table 1). The reasoning behind this method is that high values of the autocorrelation coefficient are indicative of less independence between sample points, and therefore longer consecutive sequences are required for an overall significant effect than in cases of independent samples. In our case the autocorrelation values, averaged across participants, in the different conditions for the difference signals (deviant−standard) in the test interval (120–240 ms) typically fell in the range between *Φ* = 0.7 and *Φ* = 0.9; given our design of *N* = 10 subjects, *T* = 24 samples, and *p* = 0.05, this corresponds to required sequence lengths of at least 4–6 consecutive points with *t*-values below the 0.05 significance.

Having identified a MMN effect in the time interval of 160–200 ms using the method described above, we examined if the amplitude of the MMN differed between conditions. To this end, we performed a two-way repeated measures *analysis of variance* (ANOVA) with *conserved feature* (SizeCon vs. LumCon) and *presentation side* (Left vs. Right) as factors. As input data for this analysis we used the average amplitude values of the detected MMN, i.e., the difference in ERP amplitudes (deviant condition−standard condition) averaged in the time interval from 160 ms to 200 ms.

#### Time Frequency Analysis

In addition to the classical ERP analysis described above, we also performed a TFA to determine frequency-dependent effects that might not have been accessible in the time domain. To prepare the data for the TFA, each trial was first baseline-corrected for the time interval −200 ms to −100 ms. Then each trial was transformed with a continuous complex Morlet-wavelet-transformation (three Morlet parameters) for the frequency range 1–40 Hz (which was within the previously applied filter range) with 40 logarithmic steps and for the full time range (−200 ms to 500 ms). Additionally, uniform scale power (unit energy normalization) wavelet normalization was applied. As a result, a 40 × 700 array (hereafter named TFA-array) of positive spectral amplitude values [μV] was computed from each trial. The resulting TFA-array contains oscillatory components that are not phase locked to the stimulus presentation and thus might cancel out in the trial-averaged ERPs (so called induced oscillations); however, it also contains the time-frequency response of the trial-averaged ERP itself (called evoked oscillations). In order to separate evoked and induced components, we calculated the time-frequency response of the trial-averaged ERP yielding the evoked components only (evoked TFA). We then subtracted the evoked components from the TFA-array, that contained both the induced and evoked components, resulting in data that only contained the induced effects (induced TFA). Based on results from another vMMN (Stothart and Kazanina, 2013) and an auditory MMN study (Fuentemilla et al., 2008), we expected early effects in the theta band followed by later effects in the alpha band. As the time windows we had determined in our ERP analysis (see above) were comparable to those reported by Stothart and Kazanina (2013), we used the same time windows as determined in the ERP analysis in our statistical analysis of the results of the TFA. For both, the evoked and the induced TFAs, we averaged the TFA responses from 160 ms to 200 ms within the theta band (4–8 Hz) and from 325 ms to 375 ms within the alpha band (8–12 Hz), separately for the deviant and standard condition for each participant and condition. On these data we performed three-way repeated measures ANOVAs with *deviance, presentation side* and *conserved feature* as factors.

## Results

Data were obtained from 10 participants, performing a total amount of 64,800 trials in each condition of conserved feature (SizeCon and LumCon). A total of 26.6% (*n* = 34,482) of the trials had to be rejected from further analysis either due to being a response trial, or because of button presses or other artifacts (for details, see “Materials and Methods” Section). This procedure left 47,536 trials (22.0% of them deviant trials) in condition SizeCon and 47,582 trials (21.9% of them deviant trials) in condition LumCon. On average each participant performed 2,378 valid trials (std: 313) per condition (SizeCon vs. LumCon, Left vs. Right). Task performance of all participants was high: on average 89.9% of the fixation point changes were detected (std: 6.6%). False positive reports occurred solely in 0.4% (std: 0.3%) of all trials.

### Spatial Distribution of ERPs

**Figure 3:**
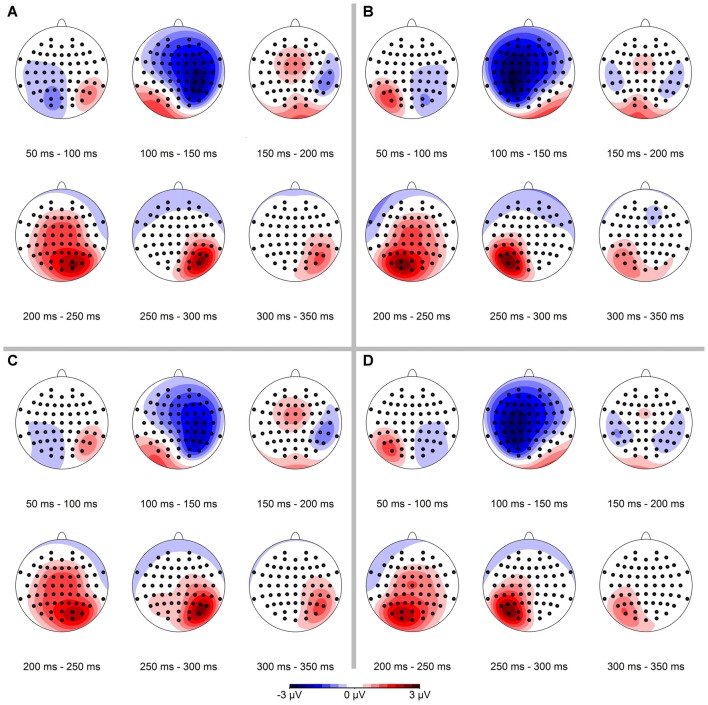
**Spatial distribution of event-related potential (ERP)-signals, separated for conserved feature** (LumCon: **A,B**; SizeCon: **C,D**) and stimulus presentation position (Left: **A,C**; Right: **B,D**). ERPs were binned in 50 ms time intervals from 50 ms to 350 ms. Scale is from −3 μV (blue) to 3 μV (red). The N1-component occurs in the time interval from 100 ms to 150 ms on contralateral parieto-frontal electrodes in all four conditions. In addition a positive component (P2) occurs ipsilateral on parietal electrodes in the time interval from 200 ms to 300 ms.

In a first step we analyzed the spatial distribution of the ERPs. The resulting data are shown in Figure 3, separately for conserved feature as well as stimulus presentation side. In the data analysis, we binned mean ERPs in 50 ms bins in the time interval between 50 ms and 350 ms. The N1-component occurred mainly in the interval from 100 ms to 150 ms contralateral to the visual hemifield the stimulus was presented in.

**Figure 4:**
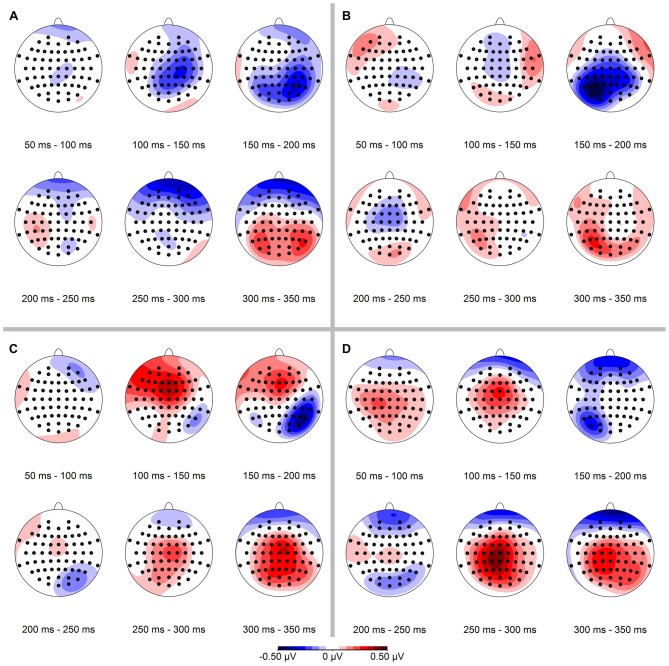
**Spatial distribution of ERP-differences (condition deviant−condition standard), separated for conserved feature** (LumCon: **A,B**; SizeCon: **C,D**) and stimulus presentation position (Left: **A,C**; Right: **B,D**). ERPs were binned and averaged in 50 ms time intervals from 50 ms to 350 ms. In all conditions, the mismatch negativity (MMN) occurred mainly in the time interval from 150 ms to 200 ms on parieto-occipital electrodes contralateral to the side of stimulus presentation.

In a next step we calculated the spatial distribution of the ERP-difference between deviant and standard condition, again separately for conserved feature as well as stimulus presentation side (see Figure 4). Mean ERP-differences were binned in 50 ms time intervals between 50 ms and 350 ms. In line with our hypothesis we observed a negativity in the ERP-difference response on parietal-occipital electrodes contralateral to the stimulus presentation side. In the binned data this effect was most pronounced in the time interval from 150 ms to 200 ms.

### Response Dynamics

Qualitatively, the spatial distribution of the ERPs supported our hypothesis of a MMN concerning numerosity to occur on the parieto-occipital electrodes contralateral to the side of stimulus presentation, i.e., electrodes P6, P8, PO8 and P5, P7, PO7 for left hemifield and right hemifield stimuli, respectively. For a quantitative analysis of the time course of the effect we focused our further analysis on the ERPs from these electrodes. Since the ERP amplitudes were first averaged for each stimulus number and then across all numbers, each number had an equal contribution to the results. As described above, each deviant stimulus also served as standard stimulus in another condition, so that physically identical stimuli could be compared.

**Figure 5:**
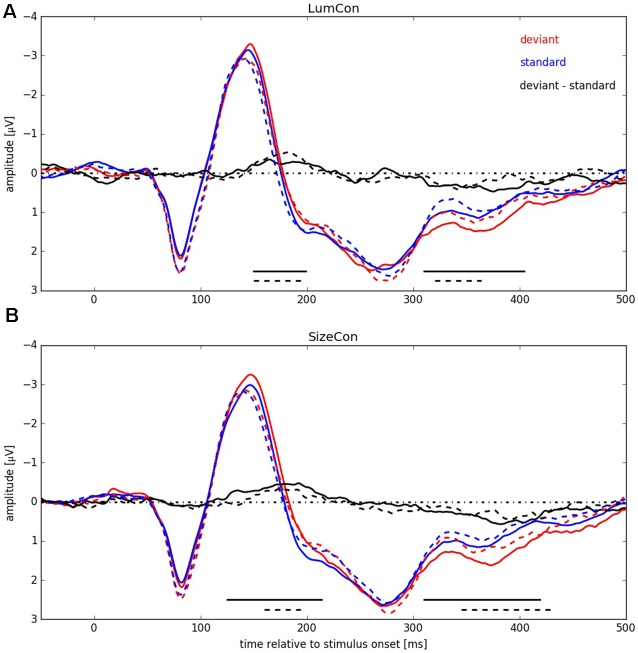
**ERP signals pooled over all participants for both conserved features** (LumCon: **A**; SizeCon: **B**). ERPs are shown in red (deviant condition) and blue (standard condition). Difference between ERPs (deviant condition—standard condition) is shown in black. ERPs from conditions with stimulus presentation side Left (averaged over electrodes P6, P8 and PO8) are shown in continuous lines (dashed lines for Right, averaged over electrodes P5, P7 and PO7). Amplitudes [μV] are plotted over time relative to stimulus onset [ms]. Black lines at the bottom indicate significant negative differences between deviant and standard condition.

In a next step we pooled the ERPs across subjects within the four conditions (*LumCon × SizeCon*) and (*Left × Right*) (see Figure 5). For both conserved features a P1-component in the time interval from 50 ms up to 105 ms with a peak at about 80 ms and a N1-component in the time interval from 105 ms up to 180 ms with a peak at about 145 ms was present. Following the N1 component the potentials were more negative in all of the deviant conditions compared to the standard conditions, resulting in a negative difference wave (ERP deviant−ERP standard) between 150 ms and 200 ms (MMN). Furthermore, the P2-components (180–400 ms with a peak at around 280 ms) differed between deviant and standard condition. The deviant condition was more positive resulting in a positive difference for times beyond 310 ms for all four conditions. This effect presumably corresponds to a P3a component that is assumed to reflect automatic processing of unexpected stimuli as part of an orienting reflex which is often following a MMN response (e.g., Escera et al., 1998, 2000).

We tested for significant differences between deviant and standard ERPs by applying the method introduced by Guthrie and Buchwald (1991). To this end, we calculated single-sided *t*-tests, separately for stimulus presentation positions and conserved feature, thereby testing the hypothesis that the potential difference between amplitudes in deviant and in standard conditions was statistically significant and below zero, i.e., negative (for details, see “Materials and Methods” Section). The analysis revealed for condition LumCon for both presentation sides that differences in 11 continuous samples between 150 ms and 220 ms were significant and below zero. For condition SizeCon Left, differences in 18 continuous samples between 125 ms and 214 ms, and for SizeCon Right in seven continuous samples between 160 ms and 194 ms were statistically significant and below zero. First order autocorrelation of ERP differences in the investigated time interval (120–240 ms) were *Φ* = 0.84 (Left) and *Φ* = 0.91 (Right) for condition LumCon, and *Φ* = 0.82 (Left) and *Φ* = 0.86 (Right) for condition SizeCon. According to Guthrie and Buchwald (1991, their Table 1) and given the settings in our experiment (*N* = 10 subjects; *T* = 24 samples; *p* = 0.05) a minimum of six continuous significant samples would have indicated a significant difference between deviant and standard ERPs in case of our autocorrelation values. Hence, the observed MMN was statistically significant in all four conditions.

Having detected a MMN, we aimed to determine if the amplitude of the effect was different between the conditions *presentation side* (Left vs. Right) and *conserved feature* (LumCon vs. SizeCon). To this end, we calculated the average MMN amplitude within the 160–200 ms time interval separately for every condition. Using these values we performed a two-way repeated measures ANOVA with *presentation side* (Left vs. Right) and *conserved feature* (LumCon vs. SizeCon) as factors. We did not observe a main effect for either of the factors (all *F*_(1,9)_ < 0.1, *p* > 0.85) or an interaction between the factors (*F*_(1,9)_ < 3.4, *p* > 0.09). It is important to note that this finding does not imply the absence of a visual hemifield effect. Such a key effect of visual hemifield is indeed presented in Figure 4. But since the ANOVA was performed on data of the chosen electrodes (Left: P6, P8, PO8; Right: P5, P7, PO7) this key effect of visual hemifield was removed. Hence, in the ANOVA the finding of “no main effect of presentation side” reflected that there was no significant lateralization of MMN that exceeded the above described key effect of the anatomically lateralization of presentation in different visual hemifields.

Beyond the main hypothesis of our study concerning the MMN, it was noticeable that the ERPs showed differences in the P300 component (300–425 ms) in a way that deviant responses had a higher amplitude than standard responses (see Figure 5). Therefore, we tested the difference in this time range with the method introduced by Guthrie and Buchwald (1991), as described before with the modification that, since we had no previous expectations of this difference, a two-sided *t*-test was used instead of the single-sided *t*-test used for MMN-analysis. First order autocorrelation (Φ) of all four conditions ranged from 0.79 to 0.86. The test-interval contained 30 samples so that, according to Guthrie and Buchwald (1991, Table 1), nine significant samples would have been required to indicate a significant effect. In all four conditions between nine and 22 significant consecutive samples were found, resulting in significant effects for LumCon Left (310–404 ms), LumCon Right (320–364 ms), SizeCon Left (310–419 ms) and SizeCon Right (345–429 ms).

### Time-Frequency-Analysis

**Figure 6:**
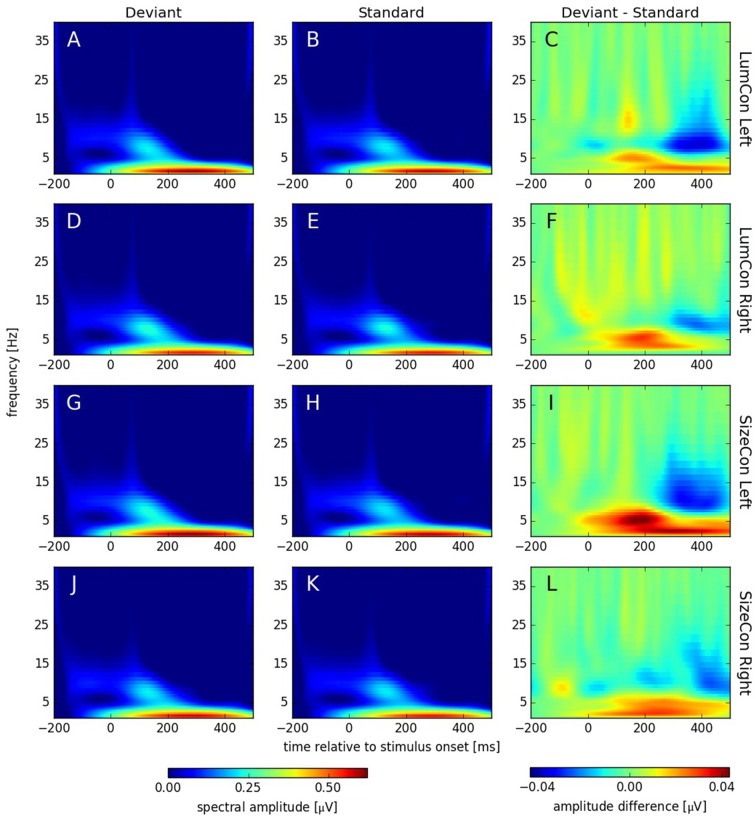
**Spectral amplitudes [μV] of continuous complex wavelet transformation (time-frequency-analysis [TFA]) plotted for time [ms] at the abscissa and frequency [Hz] at the ordinate.** The first column **(A,D,G,J)** shows responses to deviant and the second column **(B,E,H,K)** responses to standard stimuli. The third column **(C,F,I,L)** displays the difference (deviant—standard) of both conditions. The rows correspond to the presentation conditions: LumCon Left **(A–C)**, LumCon Right **(D–F)**, SizeCon Left **(G–I)** and SizeCon Right **(J–L)**.

Recent studies have shown that MMN responses contain oscillatory components in the theta and alpha band of the EEG (4–8 Hz) that might be omitted in classical ERP analysis, but can be revealed by TFA (Fuentemilla et al., 2008; Stothart and Kazanina, 2013). TFA is a method that is complementary to ERP analysis and has the potential to also reveal oscillatory effects that are not phase-locked to the stimulus and thus might cancel out when averaged across trials. Hence, this analysis shows not only stimulus evoked (i.e., phase-locked to the stimulus) but also stimulus induced (i.e., non-phase-locked) responses (see Herrmann et al., 2014). Using complex Morlet-wavelet-transformations on the single trial data, we first obtained data that contained both evoked and induced components (see Figure 6). After stimulus onset, all conditions showed increased activity in the delta (1–4 Hz) and alpha band (8–12 Hz) with peaks between 200 ms to 400 ms and 100 ms to 200 ms, respectively. The difference plots revealed an enhancement in the theta band (4–8 Hz) at around 175 ms and a reduction around 350 ms in the alpha band in all conditions. To investigate whether these effects were caused by oscillatory processes that are phase locked to the stimulus presentation or not, we separated the evoked and the induced components of the TFA (see “Materials and Methods” Section) and calculated for both cases a three-way ANOVA with *deviance, presentation side* and *conserved feature* as factors. According to the time windows for which we had observed significant ERP effects (see above), we pooled data from 160 ms to 200 ms for the theta and from 325 ms to 375 ms for the alpha band effect. In the theta band, we found a significant main effect for the factor *deviance* (*F*_(1,9)_ > 6.7, *p* < 0.03) for the evoked, but not for the induced data (*F*_(1,9)_ < 0.35, *p* = n.s.); all other effects were not significant in both cases (all *F*_(1,9)_ < 3.36, *p* > 0.1). In the alpha band, we found significant main effects for the factor *deviance* for the evoked (*F*_(1,9)_ > 14.5, *p* < 0.01) and the induced data (*F*_(1,9)_ > 10.1, *p* < 0.012). All other effects were not significant (all *F*_(1,9)_ < 4.1, *p* > 0.74).

## Discussion

We have performed a vMMN experiment on numerosity. Across conditions, displays with different numbers of circular patches (1, 2 or 3) served as standard and as deviant stimulus, so that standard and deviant stimuli differed solely in presentation frequency. Participants were engaged in a difficult perceptual task at the fixation point drawing their attention away from the stimuli presented in the visual-field periphery. Low-level stimulus features (patch size and absolute luminance) were held constant in two separate conditions. Our results show clear vMMN responses in all conditions revealing a preattentive component of numerosity processing in the subitizing range.

### Preattentive vs. Attentive Processing of Numerical Information

While subitizing has usually been considered a preattentive process (Trick and Pylyshyn, 1993, 1994; Pylyshyn, 2001), more recent studies have argued that it is influenced by attentional load (Olivers and Watson, 2008; Railo et al., 2008; Anobile et al., 2012). Our results show that task-irrelevant stimuli, comprised of a number of patches that lies well within the subitizing range, are processed in a way that evokes MMN responses, an established electrophysiological marker for preattentive processing. In view of this result, the question arises how these seemingly different findings might be reconciled?

First of all, it is important to keep in mind that the approaches in our and in previous studies relied on different definitions of preattentive processing. We, as well as the vast number of other MMN studies (for a review, see e.g., Paavilainen, 2013), reason that, if non-attended, task irrelevant stimuli evoke a MMN response, they must have been subject to some sort of preattentive processing. Behavioral studies typically test if certain measures of performance, like reaction times or discrimination thresholds, are influenced by attentional load. Initially, the notion of subitizing being a preattentive process has been mainly based on behavioral findings. In particular, in enumeration tasks reaction times are fast and increase only slightly with increasing number of display items within the subitizing range, suggesting largely parallel processing of the individual items (Trick and Pylyshyn, 1993, 1994). For larger set sizes, in contrast, reaction times are longer and show steeper increases, characteristic for sequential processing. Trick and Pylyshyn (1994) proposed a widely recognized model that is able to account for these findings. In their notion, enumeration is considered a multi stage process in which items are pre-processed in parallel in an initial, capacity limited preattentive stage which is followed by subsequent serial processing that is susceptible to attentional modulations. This is certainly a rather abstract description of the actual neuronal mechanisms that are certainly more convoluted. However, it points to the problem that unless a complete isolation of the two stages is realized, the measured data will still contain contributions from both stages (for related discussions, see Trick and Pylyshyn, 1994; Olivers and Watson, 2008). It is possible that this reasoning applies to the results of the behavioral studies that found attentional modulations in the subitizing range. On the other hand, the MMN response might have more direct access to the preattentive processing stage, potentially because it does not explicitly involve the subjects in a numerical task. Furthermore, it is currently still debated, whether large and small numerosities are actually processed by the same underlying neuronal system or whether multiple, potentially overlapping or supplementary, mechanisms exist for the different ranges (see Feigenson et al., 2004; Hyde, 2011 for reviews). Single cell recordings in the macaque monkey have identified a population of neurons in the PPC that encodes a continuous range from small to large numbers (Nieder, 2005). Given the functional similarities of the visual systems in humans and non-human primates, it is likely that such a mechanism exists in humans as well. In our current study we measured the vMMN for stimuli in the subitizing range only; consequently, we cannot draw any conclusions as to whether vMMN responses would also occur for higher numerosities. Importantly, this also implies that it is quite possible that the vMMN as observed in our study was actually caused by a general mechanism that operates across the complete number range, rather than by a genuine system that is specific to the subitizing range. In line with this, Anobile et al. (2012) have recently reported that estimation of large numerosities is less prone to attentional modulations than subitizing.

### The Role of Oscillatory Processes

In addition to the ERP analysis, we performed a TFA to investigate whether the MMN was accompanied by additional oscillatory processes. In line with another vMMN study (Stothart and Kazanina, 2013), we found a reduction in induced oscillations only in a late time window (in the alpha band), but no induced effects in the time window during which the MMN was observed in the ERPs (i.e., from 160 ms to 200 ms). In contrast to Stothart and Kazanina (2013) we observed a significant increase in evoked theta-power in this early time window; an auditory MMN study (Fuentemilla et al., 2008) reported an increase in evoked theta power for the frontal, but not for the temporal component of the auditory MMN. Clearly, more studies are needed to understand the contributions of oscillatory processes to the MMN.

### The MMN and Low Level Visual Features

A prerequisite in all MMN experiments is the control of low level stimulus features. This is important because, for example, red stimulus patches elicit ERPs that are different from those of green patches (Klistorner et al., 1998); therefore, the difference in ERPs between red standard and a blue oddball stimuli does not only contain potential MMN responses, but also reflects the trivial differences in the ERPs due to the different low level features of the stimuli.

In our study controlling low level features was also important because a number of studies have reported effects of numerosity on various ERP components by now (Libertus et al., 2007; Hyde and Spelke, 2009, 2012; Park et al., 2016). In particular, Hyde and Spelke (2009) examined dot patterns with small (numbers 1, 2 and 3) and large numbers of items (numbers 8, 16 and 24) in a passive viewing paradigm. They found that N1 components (140–175 ms) for small numbers became more negative with increasing number magnitude, while for large numbers the opposite was true. A decrease in N1 amplitude with increasing number of dots for small set sizes has also been shown when participants were actively involved in a number comparison task (Libertus et al., 2007).

In our study, we followed the common practice to compare stimuli that were physically identical and only differed because they served as standard in one condition and as oddball in the other. That is, we did not compare ERPs from stimuli that contained, for example, two patches to those of three patches. Instead, we only calculated response differences between stimuli of equal numerosity; again, these stimuli only differed because they had been presented in one sequence as a standard and in a different sequence as an oddball. Because of this control, our results can neither be explained by differences in the ERPs between standards and oddballs caused by low level visual features, nor by the reported effect of a decreasing N1 component with increasing number. We therefore conclude that our results reflect genuine MMN responses.

It is important to note, however, that it is not possible to control for all low-level-features at the same time in a sequence of stimuli that contain different numbers of objects. For example, increasing the number of circular patches while keeping the radius of the patches constant leads to an increase in overall luminance. On the other hand, keeping luminance constant requires an appropriate adjustment in patch size (more patches are on average smaller) or patch luminance (more patches are less luminant). Similar arguments can be made for the *total* circumference or the density of the patches. To cope with this problem, we decided to measure two different conditions in our experiments. In one condition the overall luminance induced by all stimuli was held constant within a stimulus sequence (LumCon); as a consequence, however, patch size was decreasing with increasing number of patches in this condition. In the second condition, dot size was held constant, thereby increasing overall luminance with increasing number of patches. Hence, we cannot exclude the possibility that in one condition the change in luminance and in the other condition the change in patch size elicited a vMMN. A hint that this might not have been the case is that the spatial distribution of ERPs in both conditions was very similar. Further evidence for this comes from a recent study on how various low level features modulate ERPs in comparison to numerosity (Park et al., 2016). Park et al. (2016) systematically varied multiple visual properties (individual and total item area, individual and total item perimeter, field area, and sparsity) together with numerosity. Using a regression based approach they could show that ERPs are actually more sensitive to changes in numerosity than to any of the other tested properties. Similar to our findings, this effect was most pronounced bilaterally over parieto-occipital electrodes and peaked at 180 ms, i.e., within the same time window in which we observed the MMN (150–200 ms). Even though this co-occurrence in space and time might be a coincidence, we think it is a likely possibility that the neuronal sources that caused the MMN we observed here are similar to those that carried the effects described by Park et al. (2016) and, rather than being a consequence of patch size or luminosity, reflect a genuine response to numerosity.

### Lateralization of the MMN

Since the presentation of the stimuli was lateralized (stimuli were located only in the left or the right half of the visual field), we expected the strongest MMN responses contralateral at parietal electrodes (see Plodowski et al., 2003; Libertus et al., 2007; Hyde and Spelke, 2009; Hyde and Wood, 2011). Our data were in line with this hypothesis (see Figure 4). The repeated measures ANOVA of our data showed no significant effect of presentation side on MMN amplitude. It is important to note that the clear effect of stimulus presentation in different visual hemifields (see Figure 4) was removed before this ANOVA due to the selection of different electrodes for different presentation sides (see “Materials and Methods” Section). Hence, the results of the ANOVA that revealed no main effect of response side indicates that there was no difference of the MMN between the response sides that exceeded the beforehand assumed lateralization due to a hemifield effect. In other words, the vMMN occurred lateralized (in right parietal electrodes for presentations in the left visual hemifield and vice versa) but the MMN did not differ between the two hemifields.

## Conclusion

We performed a visual oddball experiment to investigate the vMMN in response to changes evoked by visual stimuli of small numerosity (1, 2, or 3 patches). Even though the stimuli were task-irrelevant and the attention of the participants was drawn to a different location, the stimuli elicited robust MMN responses in all conditions. We consider this as evidence that the processing of visual numerical information in the tested number range has a preattentive component. Future studies will be needed to investigate whether this preattentive component is confined to the subitizing range or whether it is a general feature that occurs across a larger range of numbers.

## Author Contributions

The study was planned by PNH, SK and FB. The experiment was designed and programmed by PNH. Data were collected by student research assistant Natalie Heyse, CS and to the most part by PNH. Data were analyzed by PNH. The manuscript was written by PNH (including the first full draft), CS, SK and FB.

## Conflict of Interest Statement

The authors declare that the research was conducted in the absence of any commercial or financial relationships that could be construed as a potential conflict of interest.
